# Imitation as a mechanism in cognitive development: a cross-cultural investigation of 4-year-old children’s rule learning

**DOI:** 10.3389/fpsyg.2015.00562

**Published:** 2015-05-13

**Authors:** Zhidan Wang, Rebecca A. Williamson, Andrew N. Meltzoff

**Affiliations:** ^1^Department of Psychology, Georgia State University, Atlanta, GA, USA; ^2^Institute for Learning and Brain Sciences, University of Washington, Seattle, WA, USA

**Keywords:** imitation, rule learning, weight, categorization, cross-culture, social learning

## Abstract

Children learn about the social and physical world by observing other people’s acts. This experiment tests both Chinese and American children’s learning of a rule. For theoretical reasons we chose the rule of categorizing objects by the weight. Children, age 4 years, saw an adult heft four visually-identical objects and sort them into two bins based on an invisible property—the object’s weight. Children who saw this categorization behavior were more likely to sort those objects by weight than were children who saw control actions using the same objects and the same bins. Crucially, children also generalized to a novel set of objects with no further demonstration, suggesting rule learning. We also report that high-fidelity imitation of the adult’s “hefting” acts may give children crucial experience with the objects’ weights, which could then be used to infer the more abstract rule. The connection of perception, action, and cognition was found in children from both cultures, which leads to broad implications for how the imitation of adults’ acts functions as a lever in cognitive development.

## Introduction

The ability to learn from others’ actions sets our species apart. Human infants and toddlers have a proclivity, rare in the animal kingdom, for imitating a broad range of acts (Meltzoff et al., 2009; Whiten et al., 2009). This includes reproducing not only the overall outcome or endstates that others achieve with objects, but also the precise means used to attain them. For example, after witnessing the novel act of an adult touching a light panel with his head to illuminate it, 18-month-olds are likely to perform this novel act even after a 1-week delay (Meltzoff, 1988). The neural basis for infant and childhood imitation is being uncovered using electroencephalography (EEG; Marshall and Meltzoff, 2014).

Imitation has several advantages for cognitive development. Reproducing others’ precise actions accelerates and supports cultural learning of instrumental actions and arbitrary rituals (Tomasello, 1999; Boyd and Richerson, 2005; Meltzoff et al., 2009; Herrmann et al., 2013). Instrumental innovations and social routines can spread through communities through imitation, thereby leading these behaviors to be maintained across generations and providing more opportunities for cumulative progress.

A particular benefit of high-fidelity imitation is that it increases learning opportunities (Williamson and Markman, 2006). Even if acts are not fully understood, children who are able to imitate them in precise detail gain opportunities to discover a deeper meaning and cognitive understanding of the acts, which are first grasped only in a more superficial manner. In this paper, we hypothesize that action imitation can spark cognitive change and test this idea with a novel procedure using the categorization of objects by their weight. We conducted these tests in two cultures, China and the USA.

What children learn from others’ actions is not limited to specific observable movements. Children also infer and reproduce the goals others strive to achieve and cognitive rules that guide others’ behaviors (for review, see Meltzoff and Williamson, 2013). For example, children imitate an adult’s intended goal (e.g., Meltzoff, 1995), causal relations (Horner and Whiten, 2005; Schulz et al., 2008; Buchsbaum et al., 2011; Waismeyer et al., 2015), the organization guiding others’ acts (Whiten et al., 2006; Flynn and Whiten, 2008; Loucks and Meltzoff, 2013), and abstract rules (Subiaul et al., 2007a,b, 2014; Williamson et al., 2010; Wang et al., 2015).

Evidence for what has been dubbed “abstract imitation” comes from Williamson et al. (2010), which is the basis for the current experiment. Children in that study saw an adult sort four objects into two bins according to either a visual property, color (Experiment 1), or the sounds the objects produced when shaken (Experiment 2). When given a chance to manipulate the objects, children in the experimental groups were more likely to categorize the objects by these respective properties than were controls. The children were then presented with a generalization task—a different set of objects that differed from the originals in kind as well as in their color or the sound they produced. Although the adult never manipulated this second set, the children in the experimental group sorted these objects by the key object property (color or sound), suggesting that children learned an abstract rule that could be generalized across stimuli.

Here we extended this idea of “abstract imitation” to children’s learning about an interesting domain in physics—object weight. Categorizing by weight is a cognitively demanding task for preschool children. Results from Wang et al. (2015) show that 36-month-old children, the same age that readily learned to sort objects by their colors and sounds, were unable to learn the weight-sorting rule through observation and imitation. This finding is in line with previous research establishing that preschool-aged children struggle on tasks that require considering weights independently of object appearances (Smith et al., 1985; Schrauf et al., 2011).

Cross-cultural methods have been used to assess which aspects of social learning are culturally universal and which vary. Overall, these studies have shown substantial similarity in children’s early imitation, despite considerable differences in cultural milieu (Callaghan et al., 2011; Wang et al., 2012). For example, highly similar reactions have been demonstrated in children from an industrialized Australian city and children from remote Bushman and Aborigine communities (Nielsen and Tomaselli, 2010; Nielsen et al., 2014).

It is possible that the imitation of cognitive rules is susceptible to cultural experience, and Chinese culture presents an interesting theoretical test. China and other Asian countries have been dubbed “collectivist” cultures (Markus and Kitayama, 1991; Oyserman et al., 2002). Because of language and culture, people raised in China are thought to place relatively more emphasis on harmonious relationships than those raised in the USA and other Western cultures, dubbed “individualist” cultures. Chinese parenting practices highlight the value of groups, social cohesion, and conformity in behavior (Jose et al., 2000). Chinese society also emphasizes allowing others to save “Mian Zi” or “Face,” which commonly leads to implicit and conservative expressions of one’s opinion (Redding and Ng, 1982). Chinese child-rearing practices may provide a fertile training ground for highlighting the invisible rules and motivations that explain visible behaviors.

The current experiment tests Chinese children’s abstract imitation of rules and compares it to Wang et al.’s (2015) existing data set from American children. All children were presented with four visually-identical objects, two heavy and two light. In the Experimental group, children saw an adult heft each object and sort them (by weight) into two bins. Two control groups were used to determine what elements of the demonstration were needed to promote weight sorting. Specifically, we tested whether seeing an intentional sorting demonstration (Experimental treatment) was more effective for eliciting weight sorting than was seeing the hefting acts alone (a control for “stimulus enhancement”) or the hefting acts + the sorted endstate (a control for “emulation,” or duplicating the endstate).

One question was whether the focus on group cohesion and conformity in China may emphasize the underlying meaning of others’ behavior, which would give Chinese children an advantage in learning a non-obvious cognitive rule such as categorizing by the invisible property of weight. However, the abstract imitation of rules may be available during the early years in all cultures—a cultural universal that propels further cognitive development.

Equally important to the cross-cultural aspect, we sought to illuminate how imitation can inform theories about the relation between perception, action, and cognitive development. Past research has suggested that reproducing specific actions may prompt children to learn the underlying purpose of an act (e.g., Williamson and Markman, 2006). If this is the case, children’s imitation of the adult’s specific weighing and “hefting actions” (lifting up and down) may help them isolate and infer that underlying weight differences are the basis for categorizing the visually-identical objects. If so, it would illuminate how action imitation could foster the development of cognitive rules (see Discussion for further elaboration).

## Materials and Methods

### Participants

The participants were ninety-six 4-year-old children. Half were Chinese (*N* = 48; *M* = 53.06 months, SD = 3.77 months; 24 males) and half were American (*N* = 48, *M* = 48.92 months, SD = 1.66 months; 24 males). Chinese participants were recruited from a kindergarten affiliated with a university in China, which primarily enrolls children of Han ethnicity. American participants were recruited from a large metropolitan area (the sample was 78% White, 16% Black/African American, 3% other, with 2% being of Hispanic ethnicity, and 1% not reporting).

American children were tested individually in the laboratory, and their behaviors were videotaped for subsequent scoring. Chinese children were tested individually in a quiet room at their school. Georgia State University’s institutional review board (IRB) provided oversight of the project.

### Materials

**FIGURE 1:**
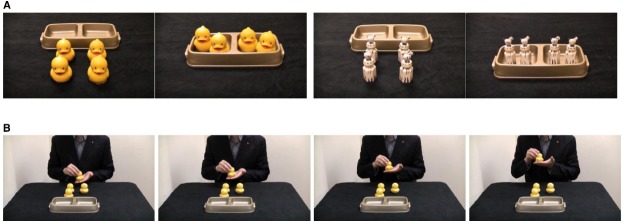
**Photographs of the experimental materials (A), which consist of sets of four visually-identical ducks, four visually-identical zebras, and bins to sort the objects into.** Within each set, two objects are heavy and two are light. Row **(B)** shows an adult making an upward “hefting” motion, which consisted of a full cycle of raising and lowering the object on a flat palm as if weighing the object.

Four sets of four objects were used as stimuli (Figure 1A). Two sets consisted of four yellow rubber ducks (5.5 cm × 4.5 cm × 5 cm) each. The other two sets consisted of four plastic zebras (5 cm × 5 cm × 4 cm). In each set, the four objects were visually identical, but unbeknownst to the child, differed in the invisible property of weight. For each duck set, two ducks weighed 87.5 g (“heavy”), and two weighed 21.7 g (“light”). For each zebra set, two zebras weighed 41.5 g (“heavy”), and two weighed 11.6 g (“light”). Pilot work suggested that the two weights used in each set were readily discriminable by untrained adults. The objects could not be discriminated by vision or audition (none of the objects made sound when manipulated, because the interior chambers were either filled or empty). The objects were spatially sorted into a two-bowled tray (23.5 cm × 5 cm × 4.5 cm), hereafter referred to as “bins.”

### Procedure

Each child was randomly assigned to one of three independent experimental groups. In all groups, the procedure consisted of a demonstration and a response period. The following three factors were counterbalanced within and between the experimental groups: (a) child’s gender, (b) the order in which the stimuli were presented (ducks or zebras as the first set), and (c) the side on which the heavy objects were placed during the demonstration (left vs. right). Each group had 16 Chinese and 16 American children.

#### Demonstration Period

***Experimental group: hefting + sorting***

The experimenter placed one set of objects (e.g., the ducks) on the table in a square arrangement (approximately 12 cm × 12 cm). The two objects of one weight were located on the right of the square, and the two objects of the other weight were on the left. The weight difference was not visible and thus unknown to the child. From the experimenter’s viewpoint, the bins were placed on the table behind the objects (Figure 1B). Then the experimenter drew the child’s attention (e.g., “It’s my turn first”).

In this group, children saw the experimenter intentionally sort the objects by weight. The experimenter picked up the object that was closest to the child (and on the child’s right), put it on his palm, and “hefted” it six times, as if to test the object’s weight by bobbing it up and down on a flat palm in a weighing motion (see Figure 1B). The object was then placed into the bin on the child’s right. Next, the experimenter picked up the second object from the child’s right side, hefted it in the same way, and placed it in the same bin. The experimenter then hefted each of the two remaining objects in the same way, and placed each of them into the other bin. The experimenter had a neutral, pleasant facial expression throughout this demonstration. The hefting motion was identical for all objects, because the experimenter practiced doing it in the same way for each object, and the difference in weight was so minimal that the kinematics of the lift could be done in the same manner.

***Control-group 1: hefting + no sorting***

In this control group, the experimenter handled each object, but did not sort them. This group was used to control for “stimulus enhancement” that may occur when the adult handles the test objects. The experimenter placed one set of objects on the table in the square arrangement, and drew the children’s attention to the objects (“it’s my turn”). Then, the experimenter picked up each object and hefted it, exactly as in the Experimental group, but instead of sorting the objects, each one was placed back on the table in its original location after it was hefted. Thus, in this control group, the children saw only the weighing process, but not the sorting behavior.

***Control-group 2: hefting + presorted***

In this control, children saw the experimenter handle each object and also saw the endstate of the objects sorted in the bins. The crucial difference was that the experimenter never sorted the objects into the bins. Instead, the four objects were brought on the table already pre–sorted into the bins. This group controls for, “emulation,” or duplication of the endstate array. The experimenter drew the child’s attention (“it’s my turn”), picked up each of the objects in turn, hefted them, and returned each to its location in the bins. Thus, for this group the children saw the weighing behavior and also the perceptual endstate that was shown in the Experimental group, but the participant never saw the adult sort the objects.

#### Response Period

The response period was identical for all groups. The experimenter placed the four objects in front of the child, with the bins behind the objects (from the child’s viewpoint, see Figure 1A). The objects were placed in a square configuration, but the two objects with the same weight were now switched (unbeknownst to the children) and placed in the horizontal rows. The spatial positioning of the objects was changed from the demonstration period so that the children had to use the object weights, and not simply the experimenter’s picking and placing movements, in order to correctly sort the objects. If children only copied the literal movements of the experimenter, they would not succeed in sorting by weight, because the array was transformed between the demonstration and response period as described. (Furthermore, the location of the heavy and light objects in the front vs. back rows was alternated for the response periods in each of the four trials. Thus if the two heavy objects were in the row closest to the child in the response period in trial 1, then they were in the row farthest from the child in trial 2, etc.)

The children were given a prompt to act, but there was no linguistic description about the content of the act. The experimenter simply made the neutral comment, “Now it’s your turn.” Children were allowed to manipulate the objects until they placed all four into the bins. If needed, the children were prompted with the question, “Can you put them inside?” After the children placed the four objects into the bins, the experimenter removed the bins for later scoring. For trial 2, the children were given an identical group of objects to sort. No demonstration was given for this set. This second set of materials was necessary because it was not always possible to score from the video with 100% certainty what the child did with the heavy/light objects, because they all were visually identical, and sometimes the child’s arm blocked a camera view; thus we retained the bins for subsequent scoring.

After these two trials, a visually novel set of four objects was introduced. If the duck set was used in the demonstration, the zebra set was used as the generalization set and *vice versa*. Crucially, these objects also differed in their absolute weights from the original (see Materials), and the experimenter did not perform any sorting demonstration with these objects. These trials were designed to assess whether children would *generalize* the weight-sorting rule to the novel stimuli. The experimenter placed the four objects of the generalization set on the table in a square arrangement (with the heavy vs. light objects in horizontal rows, see counterbalancing above) and children were given two response periods as described above.

#### Dependent Measures and Scoring

***Sorting score***

The primary dependent measure is the number of trials in which the participants sorted the four objects by weight. To be credited with a “correct sort,” children had to group the two objects of one weight in one bin and the two objects of the other weight in the other bin. Each correct sort was scored as a 1, which yields a sorting score ranging from 0 to 4 across the four trials.

***Hefting score***

Another dependent measure was also scored—children’s imitation of the hefting action that the adult had used (Figure 1B). There were three components: (a) holding the object from underneath with a flat palm, (b) hefting the object by raising the hand and letting it fall, and (c) stabilizing the object with the second hand. If children reproduced all the three components at least once in a trial, they received a score of 1 for that trial. Otherwise, the score for the trial was 0. A child’s hefting score ranged from 0 to 4 (1 possible point for each of the four test trials).

***Scoring agreement***

The primary scorer was a research assistant who remained uninformed of the participant’s group assignment and the study hypotheses. A second scorer, also unaware of group assignment, coded a randomly selected 25% of the participants. Intercoder agreement was assessed using the Intraclass correlation coefficient (ICC = 0.98). Due to IRB restrictions, videos are not available for the Chinese children. Only the American children’s hefting was scored. (In the American sample, three video records were unavailable resulting in a final *N* = 45 for the hefting analysis.)

## Results

Preliminary analyses showed no significant effects of participant sex, the side on which the weights were placed, object type (ducks vs. zebras), or presentation order (ducks vs. zebras first). We collapsed across these factors in all subsequent analyses.

### Object Categorization

**FIGURE 2:**
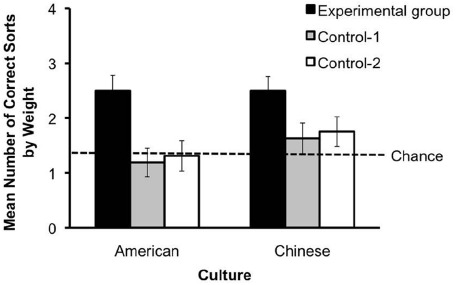
**Mean number of weight sorts (±SE) as a function of test group and culture**.

Our first analyses test for differences in whether children sorted the sets of objects by weight as a function of experimental group. Children’s sorting scores were analyzed using a 2(Culture: Chinese vs. American) × 3(Test group: Experimental, Control-1, Control-2) × 2(Object set: Demonstration set vs. Generalization set) repeated-measures ANOVA. Figure 2 shows the sorting scores as a function of Culture and Test group. This analysis revealed a significant main effect of Test group, *F*(2,96) = 9.03, *p* < 0.001, ${\text{η}}_{p}^{2}$ = 0.17. Follow-up pairwise comparisons (Student–Newman–Keuls) indicated that children in the Experimental group (*M* = 2.50, SD = 0.95) had significantly higher sorting scores than did children in either the Control-1 (*M* = 1.41, SD = 1.18; *p* < 0.001) or Control-2 (*M* = 1.53, SD = 1.19; *p* = 0.002) groups, with no significant difference between the two controls (*p* = 0.87).

This analysis also revealed several notable non-significant comparisons. Culture showed no significant main effect, *F*(1,96) = 1.91, *p* = 0.17, ${\text{η}}_{p}^{2}$ = 0.02, or interaction with Test group, *F*(2,95) = 0.48, *p* = 0.62, ${\text{η}}_{p}^{2}$ = 0.01. There was also no significant main effect of Object set, *F*(1,90) = 0.11, *p* = 0.74, ${\text{η}}_{p}^{2}$ = 0.001, or Test group × Object set interaction, *F*(2,90) = 0.33, *p* = 0.72, ${\text{η}}_{p}^{2}$ = 0.007.

There was evidence of generalization. Children’s sorting scores on the Demonstration and Generalization objects were respectively: Experimental group: *M* = 1.19, SD = 0.64, *M* = 1.31, SD = 0.69; Control-1: *M* = 0.75, SD = 0.84, *M* = 0.69, SD = 0.59; Control-2: *M* = 0.75, SD = 0.76, *M* = 0.78, SD = 0.83. No significant difference was found between children’s performance on Demonstration and Generalization objects, *t*(31) = -0.75, *p* = 0.46, *d* = 0.18, indicating that children in the Experimental group did just as well on sorting the novel objects by weight as they did in sorting the ones that the adult originally used in the demonstration–generalization. Further evidence of generalization is that 50% (16/32) of the children in the Experimental group sorted objects in three or four trials versus 20.3% (13/64) in the controls, *χ*^2^ (4,92) = 14.70, *p* = 0.005, Cramer’s *V* = 0.28.

We also conducted a more over-arching test of children’s performance. Children’s sorting scores were compared to chance. To calculate the chance value, we assumed that two objects were placed into each bin (children did this on 93.9% of trials). There are 24 possible arrangements of the four objects in the two bins. By chance combinations alone, in 8 of these 24 combinations the heavy objects would be grouped together in one bin and the light objects in the other bin. Thus, the chance probability that the final array will consist of two objects of the same weight placed in each bin is 0.33. Considering that there are four trials, chance performance is a sorting score of 1.33 (4 trials × 0.33). A one-sample *t*-test revealed that children in the Experimental group categorized the objects by weight significantly more often than is expected by chance, *t*(31) = 6.96, *p* < 0.001, *d* = 2.50. In contrast, children’s performance in the Control-1 (*p* = 0.72) and Control-2 (*p* = 0.35) groups was not significantly different from chance. This same effect was also obtained for the Chinese and American cultures tested individually.

### Hefting Behavior

This analysis assesses whether children imitated the specific “hefting” act and how this interacted with their learning the cognitive rule of categorizing the objects by weight. This question is of interest because one way that children could learn about weight is by imitating the motor acts of hefting (bobbing the object up and down in the hand while supporting it), even if they did not fully understand why the adult was doing this act. In this way, imitation of the motor act might potentially engender learning about the property of the object. For this analysis, we classified children across test groups into one of three sorting types based on their sorting scores. Children who correctly sorted the objects on three or four trials were considered to have a high sorting score (*high sorters*, *n* = 13). Children who sorted the objects on two trials were considered *medium sorters* (*n* = 7). Children with a sorting score of 0 or 1 were categorized as *low sorters* (*n* = 25).

**FIGURE 3:**
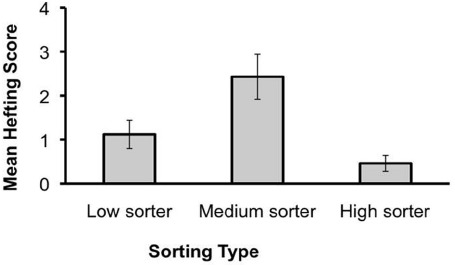
**Mean hefting score (±SE) as a function of sorting type**.

A one-way ANOVA using sorting type (three levels) as the between-subject factor was conducted on children’s hefting scores. Children’s imitation of the adult’s hefting act was related to their sorting performance, *F*(2,42) = 4.04, *p* = 0.03, ${\text{η}}_{p}^{2}$ = 0.16 (Figure 3). A follow-up pairwise test (Student–Newman–Keuls) indicated that the medium sorters had significantly higher hefting scores than did the high sorters (*p* = 0.01), with intermediate performance by the low sorters (see Discussion for further consideration).

## Discussion

Based on the adult demonstration, both American and Chinese children abstracted the categorization rule of sorting objects by weight. The low levels of sorting by the children in Control-1 (hefting + no sorting) establishes that merely seeing the adult’s weighing actions alone is not enough to induce children to categorize the objects. Control-2 (hefting + presort) establishes that seeing both the adult’s hefting gestures and the final sorted endstate is also not enough. This latter result is particularly striking and important because the behaviors used during the demonstration period of Control-2 closely trace those used in the Experimental group. In the Control-2 group, the experimenter picked up the presorted objects from the bins and returned them to the same position; in the Experimental group, the experimenter picked the objects from the table and sorted them into the bins. Neither the hefting nor the final endstate was sufficient to promote weight sorting. *We therefore suggest that the rule learning was based on the perception and imitation of the adult’s goal-directed sorting behavior*.

### Action Observation and Cognitive Rule Learning

Children who saw the experimental demonstration of categorizing visually-identical objects by the invisible property of weight showed higher rates of sorting the objects by weight than would be expected by chance. Several elements of the experimental design indicate that the children had to go beyond copying the adult’s specific motor actions alone to succeed. The spatial positioning of the heavy and light objects was switched between the adult’s demonstration and the response period. This means that if the children duplicated the literal picking up and placing movements of the adult, the objects would not have been grouped by weight. Further, the objects in each set looked identical—there were no visual cues and no auditory cues for categorizing the objects. The finding of weight sorting is in line with arguments that children’s categorization is not limited to considering only visual perceptual features, but can include the consideration of invisible and internal properties of objects (e.g., Gelman and Wellman, 1991; Gelman, 2003).

Extensive previous research has established that understanding object weight is a challenging cognitive task for children of the age tested here and even older (Piaget, 1951; Smith et al., 1985; Schrauf and Call, 2009; Schrauf et al., 2011; Povinelli, 2012). During the preschool years, in particular, children struggle to consider this internal and invisible property in the absence of correlated visible cues (Smith et al., 1985). Profound difficulties with weight have also been reported in comparative work (Vonk and Povinelli, 2006; Schrauf and Call, 2009; Povinelli, 2012). The key suggestion made in this paper is that social learning and imitation can prompt children’s attention and cognitive inferences about the invisible property of weight.

We come, then, to the crux of the problem: what exactly did children learn about weight from observing the adult’s sorting actions? One possibility is that they learned that the objects had different weights. The hefting movements used by the adult may be one cue to this invisible property. Seeing the hefting act coupled with intentional sorting behavior by the adult may have prompted children to seek an explanation for this complex behavioral stream. A good candidate explanation may be an internal, invisible property such as weight (for related discussions, see Legare et al., 2010; Legare and Lombrozo, 2014; Meltzoff and Gopnik, 2013). An additional possibility, not mutually exclusive, is that children might have already had an inkling about object weight and gained information about the adult’s goals or how to behave in this contextual situation—people sort by weight.

An important characteristic of children’s weight sorting in this experiment is that it was generalizable. The adult manipulated only the first set of objects, but the children in the Experimental group were equally likely to sort on the generalization trials. This finding highlights that rules, once abstracted, can be applied to new objects and across situations. Thus, if a child learns to consider weight when picking melons, she could also consider this invisible property in relation to other types of objects. Overall, these current findings indicate that observing the act of categorization promoted children to make use of weight with novel objects on new trials.

### Action Imitation and Cognition

Some children were more likely to imitate the hefting acts that the adult demonstrated. Children with medium sorting scores hefted the objects on significantly more trials than did children who had high sorting scores.

One function of imitating others’ hefting actions with high fidelity is that it may afford children the opportunity to discover the significance of behaviors that are not understood (Williamson and Markman, 2006). Whether or not children actually understand the deeper purpose of the hefting acts, children gain first-hand experience with the weight of the objects when they imitate the hefting behaviors. This experience may have been less important for children who readily infered the sorting rule (the high sorters)—indeed they may have realized that imitating the hefting acts was unnecessary for completing the goal of categorizing the objects by weight. However, it is possible that imitating those specific acts with high fidelity helped the intermediate sorters to attend to or recognize the weight difference and its significance, and then to use this property to categorize the objects. Although the data are too limited to draw strong conclusions, they raise intriguing links between action imitation and cognitive development—with action observation sparking action production, which may direct attention, experience, and cognitive change.

### Cross-Cultural Universals in Imitation

In China, there is generally a greater emphasis on conformity and the implicit expression of ideas than in the individualistic American culture (Markus and Kitayama, 1991; Oyserman et al., 2002). However, despite the differences in parenting practices and cultural norms, we found no difference in children’s imitation of the rules tested here. It is possible that culture exerts an influence on rule imitation that was not detected in this experiment with this specific physical-based rule (vs. a more psychological attribution). It should also be recognized that the children in both the USA and China were recruited from middle- to upper-middle class families, and with increasing globalization, it is possible that any cultural differences due to traditional child-rearing practices are not as pronounced in people of closely matched socio-economic backgrounds. Additionally, preschool children may not have had sufficient cultural experience to show differences that may emerge later; or there may be a different developmental time course for social rules and customs than for those based on physical properties such as weight. One recent example showed a different time course in the acquisition of cultural stereotypes about math in children raised in Asian vs. North American culture (Cvencek et al., 2014).

The findings of the current study are consistent with a growing body of research showing similarities in children’s imitation across a variety of cultures (e.g., Callaghan et al., 2011; Nielsen et al., 2014). Past studies have generally targeted the reproduction of specific actions on objects while the current study targeted the reproduction of an abstract cognitive rule underlying such behaviors. In early development especially, the observation and use of others’ actions may draw primarily on cultural universals. Children around the world may use imitation in similar ways to learn new, generalizable information from other social agents.

## Conclusion

The current study provides three contributions. First, it shows that children’s imitation goes beyond replicating specific motor movements. Children also imitate abstract rules or strategies that guide behavior, such as rules for categorization. Such “abstract imitation” (Williamson et al., 2010) is important for children’s acquisition of both instrumental skills and cultural practices. Second, this research also suggests that a different type of imitation, specifically children’s high-fidelity imitation of motor acts, may serve as a lever in their acquisition of abstract cognitive rules. Children who did not understand the weight-sorting demonstration may have benefitted from reproducing the adult’s exact “hefting” acts. This use of imitation of literal behavior as a mechanism for rule learning deserves more research. Third, this research extends previous findings of cross-cultural similarity in social learning to an area beyond the imitation of particular acts to the imitation of more generalizable rules (categorization rules). Overall, these findings and others support the view that action representation and imitation may be key mechanisms for the rapid acquisition and spread of generalizable skills, knowledge, and customs in human cultures.

### Conflict of Interest Statement

The authors declare that the research was conducted in the absence of any commercial or financial relationships that could be construed as a potential conflict of interest.
